# Current status of bicompartmental arthroplasty

**DOI:** 10.1186/s42836-024-00283-x

**Published:** 2025-01-03

**Authors:** Yingjian Gao, Bing Yue, Weiwei Xin

**Affiliations:** Department of Orthopedics, School of Medicine, Renji Hospital, Shanghai Jiao Tong University, Shanghai, 201112 China

**Keywords:** Bicompartmental arthroplasty, Total knee arthroplasty, Alternative option, Functional outcomes

## Abstract

**Background:**

Bicompartmental arthroplasty (BCA) serves as a less invasive alternative to total knee arthroplasty. This review aims to present the current status of BCA.

**Body:**

Recent literature on BCA was reviewed and synthesized from the perspectives of function, radiological assessment, patient satisfaction, survival rate, patellar tracking, satisfaction survey, and revision rate.

**Conclusion:**

BCA is beneficial for patients with bicompartmental arthritis and those suffering from deterioration in other compartments after unicompartmental knee arthroplasty (UKA). Compared to total knee arthroplasty, BCA reduces trauma, accelerates recovery, and improves sports ability. While BCA is evolving towards a more exciting future, more clinical studies are warranted to exploit its potential and validate its efficacy, eventually improving outcomes and patient satisfaction.

## Background

Total knee arthroplasty (TKA) has evolved as a stepwise treatment for knee osteoarthritis (OA). However, approximately 20% of patients were dissatisfied with the outcome of TKA [1, 2], especially young patients [3]. Multiple factors contribute to patient dissatisfaction, such as failure to restore native knee kinematics, requirement for revision, and psychosocial impact [2, 4–6]. Additionally, only 33% of end-stage knee OA patients have all three knee compartments affected [7]. Partial knee arthroplasty (PKA) offers an improved alternative, especially for isolated medial compartment OA, due to its better kinematics, lower invasiveness, comparable reoperation and complication rates, lower cost, and higher cost-effectiveness [8].

Except for more than 2/3 of patients suitable for PKA, as previously mentioned, 23–28% of patients have bicompartmental involvement [7, 9, 10], potentially requiring combined partial knee arthroplasty (CPKA). Among them, 11% were suitable for medial bicompartmental arthroplasty (BCA-M), and 4% were suitable for lateral BCA (BCA-L) [7]. This review aimed to present the status of BCA.

### Indications and contraindications

Indications for BCA include pain limited to the patellofemoral and one of the tibiofemoral (TF) compartments, OA (Kellgren-Lawrence grade 2 or higher) involving the patellofemoral and one of the TF compartments, OA not more than grade 1 disease in the other TF compartment, correctable varus or valgus deformity, less than 10° fixed flexion deformity, non-significant bone loss, knee flexion of more than 90°, and intact cruciate ligaments.

Contraindications for BCA include clinical TF or patellofemoral instability, varus or valgus deformity of more than 15°, inflammatory arthritis, and patellar alta or baja.

### Classification (Terminology)

**Fig. 1:**
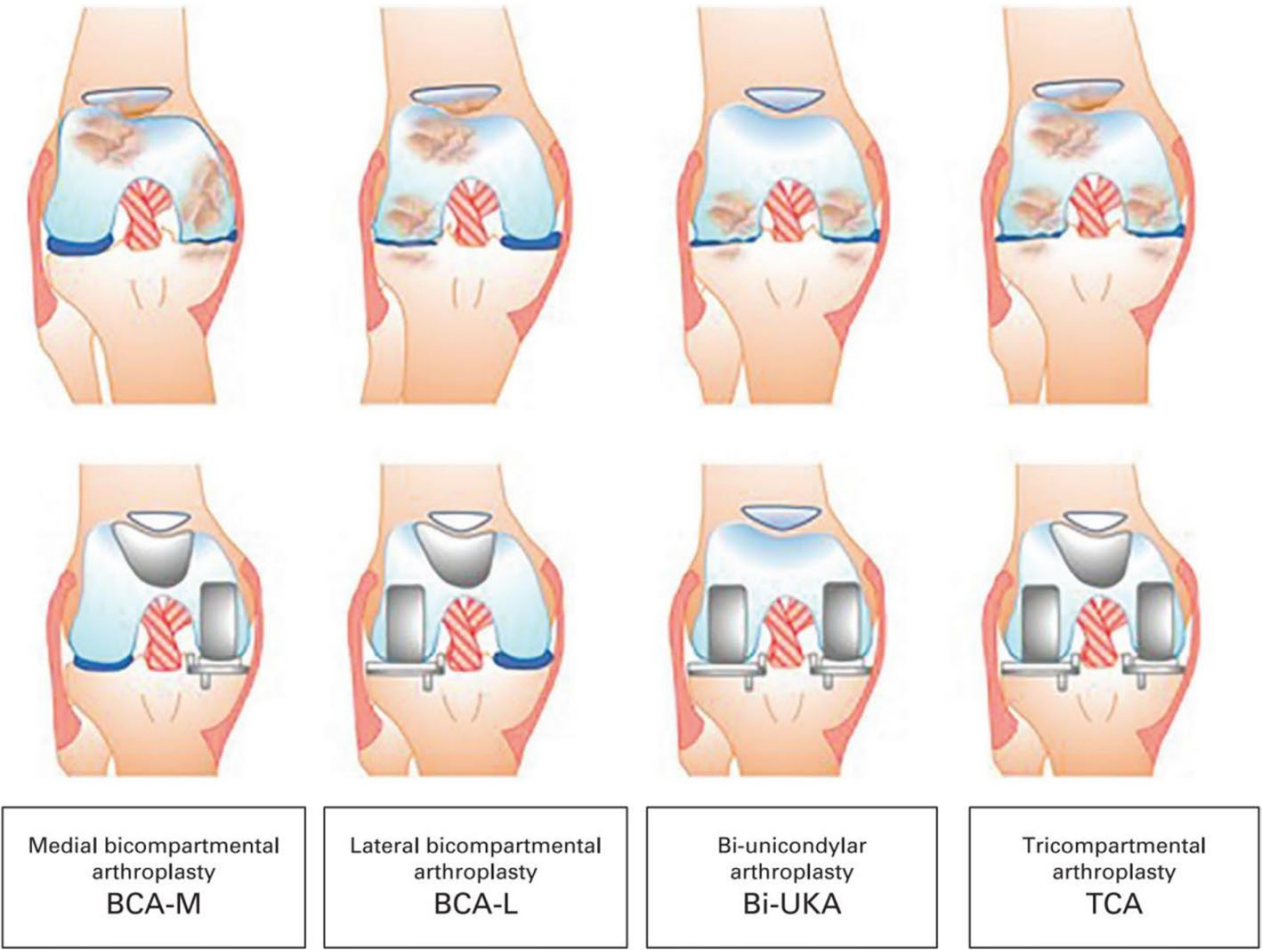
Classification of combined partial knee Arthroplasty [16]

Since the early 1970s, a combination of small implants has been employed to treat multi-compartment arthrosis, such as medial with patellofemoral arthroplasty (PFA), lateral with PFA, medial and lateral unicompartmental knee arthroplasty (UKA), and medial and lateral with PFA. Multiple terms and abbreviations have been used in the literature, such as bi-unicompartmental [11], bi-unicondylar [12], bicompartmental [13, 14], BCA [14], BKA [11], bicompartmental knee arthroplasty (BiKA) [15] and UKA + PFA [11]. To eliminate this confusion, BCA-L, BCA-M, biunicondylar arthroplasty (Bi-UKA), and tricompartmental arthroplasty are recommended for the classification of CPKA procedures according to the preferred terms of the four configurations described in Fig. 1 [16].

In terms of the prosthesis design, BCA implants can be classified into modular and monolithic types. The modular design includes a patellofemoral implant and lateral/medial UKA, which can be implanted simultaneously or sequentially. Combinations of different brands of prostheses are available [15, 17, 18].

The monolithic off-the-shelf (OTS) implant, Journey-Deuce BCA (Smith and Nephew, Memphis, TN, USA), was reportedly associated with lower outcome scores and higher revision rates, leading to its withdrawal from the market [19, 20]. Recently, a customized individually made (CIM) BCA, created using patient CT data, ConforMIS iDuo G2 (ConforMIS Inc., Burlington, MA, USA), was introduced [20–22].

### Potential advantages

The modular BCA potentially has advantages over TKA, including bone preservation, bilateral cruciate ligament preservation, minimal invasiveness, and more options for revision. The amount of bone resection in TKA is 3.5 to 4 times more than in BCA, which provides greater bone preservation [23]. Primary TKA can be done without bone grafting, augmentation, or stem when revising a failed BCA [24]. The cruciate ligaments provide better kinematics, proprioception, stability, and gait patterns while reducing friction stress between prostheses. As a minimally invasive procedure, it reduces scarring, blood transfusion rates, and operative complications [23], potentially promoting postoperative recovery. Additionally, BCA is an alternative to TKA in PKA revision for patients with subsequent second-compartment pathologies.

### Kinematics and biomechanics

Knee kinematics is influenced by ligament integrity. In the absence of an anterior cruciate ligament (ACL) when performing cruciate-retaining TKA (CR-TKA), normal knee kinematics, such as tibial internal rotation, lateral femoral rollback, and medial pivot rotation cannot be reproduced during deep flexion [25, 26]. Paradoxical anterior translation of the femur over the tibia has also been reported [26]. The simultaneous preservation of the anterior and posterior cruciate ligaments represents a higher form of leverage for the extensor mechanism, better proprioception [27], and positive axial rotation during normal walking [28], eliminating paradoxical anterior translation following TKA [29].

**Fig. 2:**
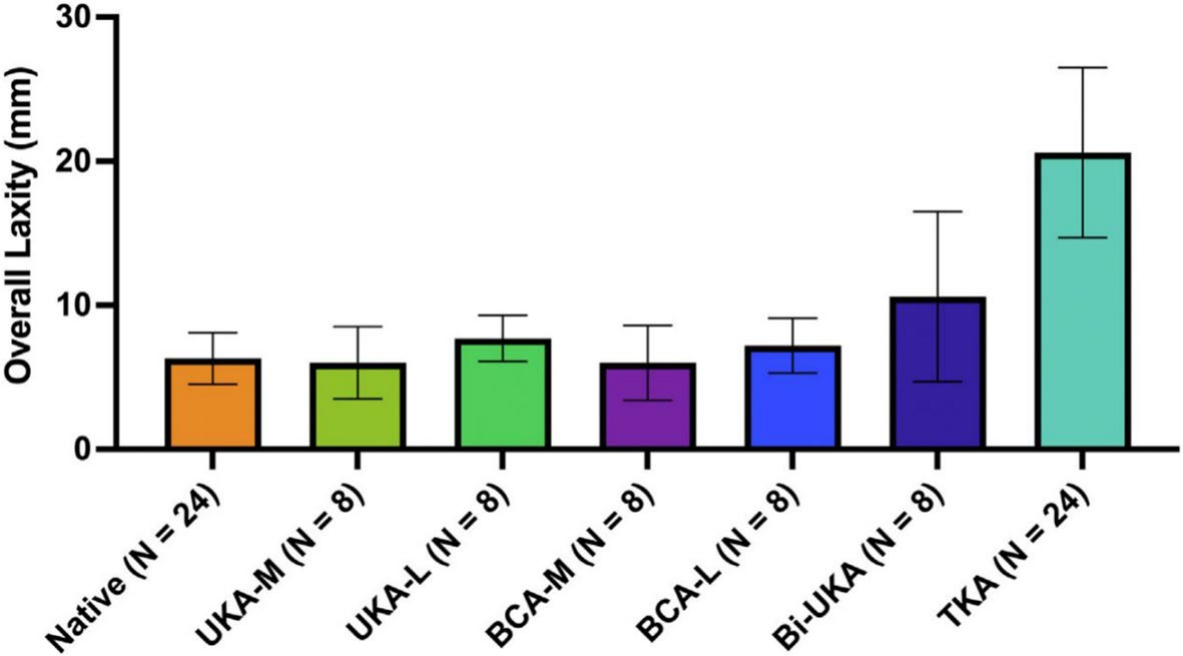
Overall anterior–posterior laxity (averaged across all flexion angles) with standard deviation for each implant state [6]

In a cadaveric study, Garner et al. focused on anterior–posterior (A-P) stability and the effects on passive ligaments after arthroplasty at 90 N of tibial anteroposterior translational force. They simulated mobile-bearing medial UKA (Oxford Partial Knee System, Zimmer Biomet, Warsaw, IN, USA), fixed-bearing lateral BCA (Oxford Partial Knee System), BCA (media/lateral UKA + Gender Solutions Patello-Femoral arthroplasty, Zimmer Biomet, Warsaw, IN, USA) and CR-TKA(NexGen Cr-Flex, Zimmer Biomet, Warsaw, IN, USA) [6]. The UKA and BCA showed laxity similar to that of the native knee, with no more than 1 mm differences with the knee flexed 0°–90°. However, CR TKA significantly increased laxity in both directions, up to 18 mm forward and 4 mm backward (Fig. 2). Functional ACL and intact menisci are the causes of the differences between BCA and TKA.

**Fig. 3:**
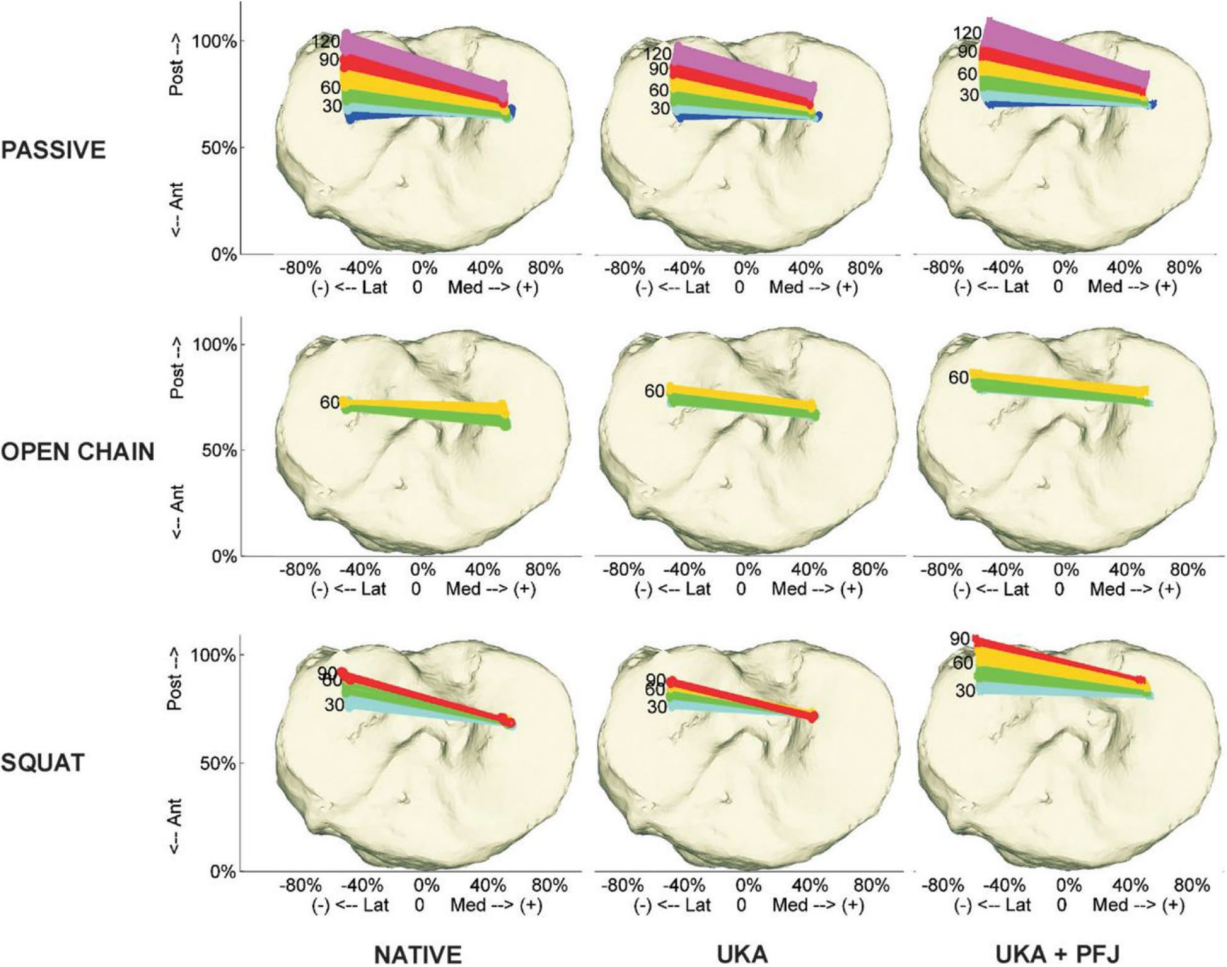
The tibial top views

Another cadaver study of a modular medial BCA used the Accuris UKA system (Smith & Nephew, Memphis, TN, USA) and an oxinium femoral component (Journey PFJ, Memphis, TN, USA) [30]. Three motion modes were simulated: passive flexion–extension cycles, open-chain extension with 3 kg of load bearing at the distal tibia, and squats at 30°–120° with a vertical ankle force of 136 N. The native knee kinematic property of the physiological femoral rollback was reproduced (Fig. 3). After medial UKA, adding the PFA led to a more posterior medial femoral condyle center, more dorsal tibiofemoral contact points, and increased MCL strain. These changes were attributed to the design of the trochlear component. Relative overfilling of the patellofemoral joint due to artificial trochlear implantation may have contributed to this phenomenon. This effect was observed despite the surgeon’s emphasis on cutting the anterior side of the femur flat to the anterior cortex to avoid a notch. This may be due to the geometry of the implant or the less compressive metal and polyethene implants replacing the compressible cartilage.

The tibiofemoral contact point remains largely unchanged with the installation of UKA in comparison to the native throughout the range of motion. BCA shifts this contact point dorsally under all testing conditions [30].

In an in vivo kinematic study, knee motion after multicompartmental knee arthroplasty (Restoris® MCK, MAKO Surgical Corp., Fort Lauderdale, FL, USA) with haptic robotic-assisted bone preparation (RIO® or TGS™, MAKO Surgical Corp) was recorded by video-fluoroscopy at an average of 13 months after surgery [31]. The 3D position of the implant components was captured during stair-walking and knee movements. Similar native tibiofemoral kinematic characteristics, such as femoral rollback and femoral external rotation, were detected in all the PKAs, including nine UKAs, three BCA-Ms, and three Bi-UKAs. The knees with the BCA-M showed the most posterior translation and femoral external translation. These results confirmed that BCA-Ms were kinematically stable with intact cruciate ligaments.

Similar results have been observed in cadaver studies [32–34]. With a functional ACL, the Journey-Deuce BCA showed rotation and anterior–posterior (AP) translation stability close to those of the native knee and superior to those of CR-TKA (GENESIS II, Smith & Nephew, Memphis, TN, USA) and PS-TKA (GENESIS II, Smith & Nephew, Memphis, TN, USA). In an in vivo kinematic study, using fluoroscopy and model image registration techniques, the monolithic implant exhibited motion patterns comparable to those of the native cruciate ligament, where the medial condyle remained relatively close to the center of the tibial plateau, while the lateral condyle rolled back with flexion [35].

With the same extension movement, lower quadriceps extension forces are considered biomechanically favorable, providing higher efficiency of the extension mechanism and better articulation constraints. Previous studies have shown that the lever arm of the extension mechanism decreases due to the paradoxical anterior movement of the femur during flexion, leading to an increase in quadriceps extension force when the ACL is deficient or non-functional [36–38]. In preserving the functional ACL, BCA-M showed better biomechanical properties. In an in vitro study, a fixed-bearing medial UKA (Sigma® PFC High Performance Partial Knee; DePuy Orthopaedics, Kirkel, Germany) and a trochlear component (Sigma® PFC High Performance Partial Knee; DePuy Orthopaedics, Kirkel, Germany) were implanted sequentially [39]. The constant extension moment of 3 Nm during the isokinetic extension cycle from 120° to 0°, represents the average extension moment throughout the isokinetic cycle, and the typical sinusoidal characteristic curve of the quadriceps force remained before and after each replacement scenario. No significant differences from the native knee were detected from 40° to 10°, suggesting that BCA-M might have advantages in knee gait kinematics. However, the anatomical alignment of the patellofemoral joint in the trochlear prosthesis may increase the maximum force of the quadriceps in deep flexion. Using the same experimental setup, the authors evaluated patellar tracking and patellofemoral pressure distribution [40]. Interestingly, they found no significant changes in patellar tracking or retropatellar area contact pressure. This supports the notion that BCA-M will result in near-physiological pressure on the patella with preservation of the physiological lever arm and femoral rollback, in stark contrast to published CR-TKA data [41, 42]. The patellofemoral contact area was significantly reduced, and the peak pressure increased, along with the effect of edge loading, which may support resurfacing the patella and correcting the implantation without overhang.

**Fig. 4:**
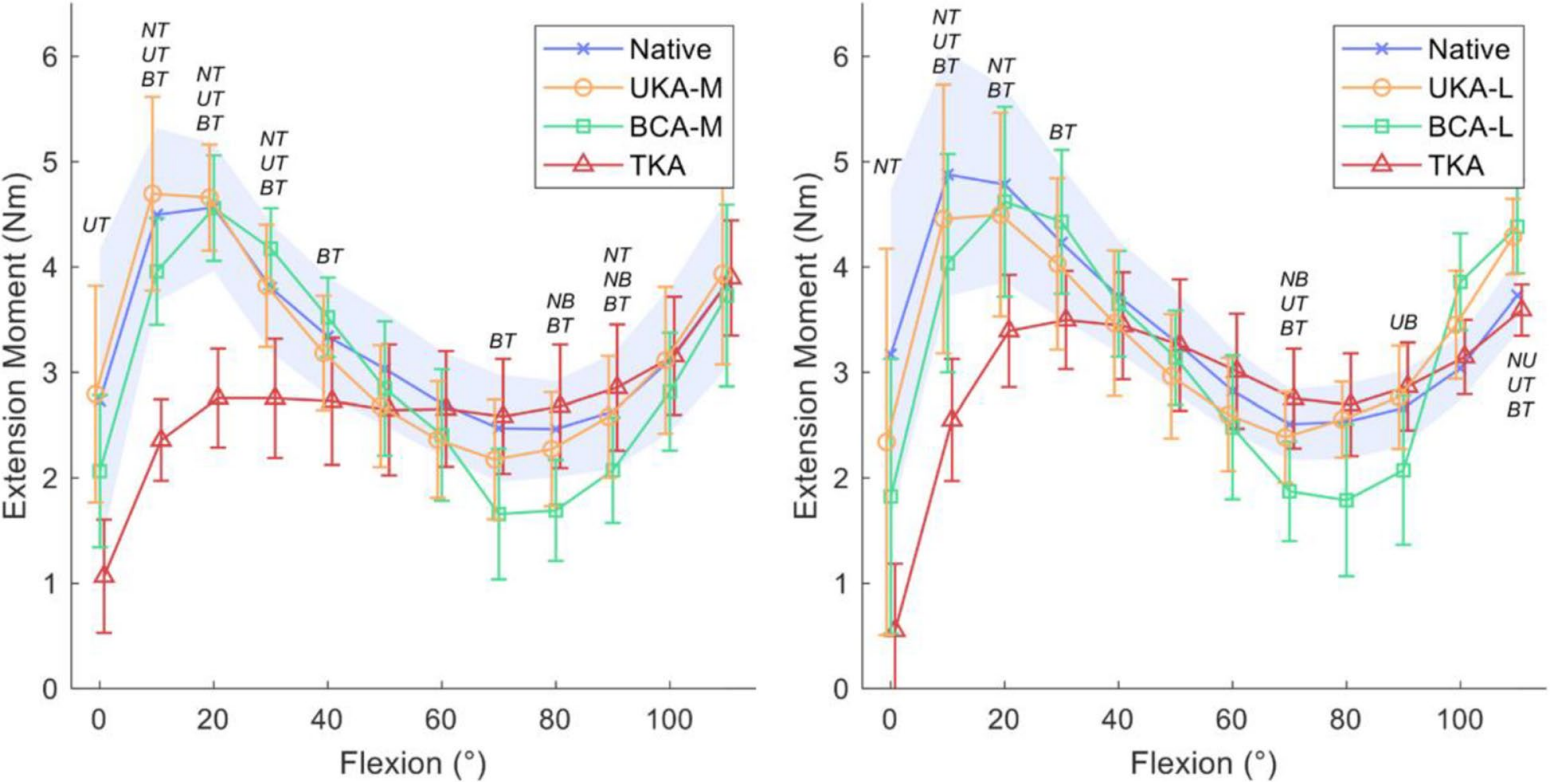
Comparison of extension moment-flexion angle relationships among native knees and different arthroplasty types. (Static flexion angles against mean extension moment (Nm) for native knees, lateral unicompartmental knee arthroplasty (UKA-L), lateral bicompartmental knee arthroplasty (BCA-L), and total knee arthroplasty (TKA); 95% confidence intervals with a shaded blue area for the native knee, and bars for implanted knees. Italicized letters indicate pairwise statistical differences (*P* < 0.05): NU, native vs. UKA-L; NB, native vs. BCA-L; NT, native vs. TKA; UB, UKA-L vs. BCA-L; UT, UKA-L vs. TKA; BT, BCA-L vs. TKA [43])

Garner et al. fixed the loading weight and traction direction of the quadriceps femoris, iliotibial band, and hamstring tendons relative to the femoral axis to determine the efficiency of knee extension from 0° to 110°. This efficiency was defined as the ratio of the energy output of knee extension through the range of flexion after arthroplasty compared to the natural knee in cadavers [43]. The study compared two configurations: the BCA-M, composed of cemented mobile-bearing medial UKA (Oxford Partial Knee Microplasty System, Zimmer Biomet, Warsaw, IN, USA) and Gender Solutions patellofemoral arthroplasty (PFA, Zimmer Biomet), and BCA-L, consisting of cemented fixed-bearing lateral UKA (Oxford Partial Knee Microplasty System, Zimmer Biomet, Warsaw, IN, USA) and Gender Solutions patellofemoral arthroplasty (PFA, Zimmer Biomet). The study found no significant difference in extension moment between the two configurations, except for a small decrease between 70° and 90° (Fig. 4). In the context of daily activity, such as the stance phase of gait (0° to 30°), stair ascent (10° to 40°), uphill walking (10° to 80°) and sit-to-stand (0° to 100°), the extensor efficiency of BCA was close to native knee. However, BCA-L was, on average, 10% ± 8% less efficient for rising movement. For CR-TKA (NexGen CR-Flex; Zimmer Biomet), the study observed significant reductions in extension moment between 0° and 30°, along with a 12% to 40% decrease in extensor efficiency for daily activities.

These data suggest that BCA retained the kinematic and biomechanical features of the native knee. This may be due to the functional ACL and meniscus in BCA, which preserves anteroposterior stability and the patellar tendon angle, allowing BCA to retain near-normal laxity and near-native extensor efficiency, unlike TKA. However, the results of these simplified models cannot be directly extrapolated to complex in vivo kinematics or physiological muscle loading conditions.

### Outcomes

The study design, prosthesis type, inclusion and exclusion criteria, patellar replacement, and outcome assessment time were heterogeneous among the different studies. However, three questions present themselves: (1) How efficacious is BCA? 2. Which is better for primary arthroplasty, BKA or TKA? (3) Which is better for revision PKA, staged bicompartmental knee arthroplasty (sBCA) or TKA?

#### How efficacious is BCA?

In general post-BCA outcomes were favorable (Table 1). Short-, mid-, and long-term follow-ups after modular BCA showed that the range of motion (ROM) and function were significantly improved, as measured on the Knee Society Score, Oxford Knee Score, Knee OA and Outcomes Score scale (KOOS), and Western Ontario and McMaster University OA Index. Similar results were obtained for the monolithic prostheses (Table 2). However, early OTS monolithic prostheses were considered ineffective compared with modular prostheses, with the problem primarily arising from femoral component malpositioning or malrotation owing to their linked design and unoptimized sizing [23, 44]. Design flaws also exist in the transition zone between the patellofemoral and femorotibial compartments, which did not account for the high variability in femoral condyle morphology [21, 44]. Third, the metal tibial tray of the Journey-Deuce BCA has been reported to have fractured in situ, leading to a product recall [45]. Finally, high failure rates have been reported [46]. CIM prostheses have yielded promising results [20–22]. Beckmann et al. [20] recruited 79 patients who were implanted with CIM-BKA (patellofemoral plus either medial or lateral tibiofemoral, iDuo G2 system, Conformis, Billerica MA) and found that the KSS score, KSS function score, and satisfaction domains significantly improved. They concluded that the score and revision rate of CIM-BKA were superior to those of monolithic OTS-BKA implants. Shamdasani et al. [21] analyzed the changes in knee alignment and the subsequent consequences of patellar tracking after CIM BKA. Among the 26 patients post-CIM BKA, 13 (50%) showed neutral leg alignment, two (8%) varus alignment, and 11 (42%) valgus alignment. Patellar tracking was central in 19 cases (73%) and lateral in seven cases (27%). Although neutral leg alignment was not restored in every case, clinical and patient-reported outcomes improved. Surgeons are concerned about the effects of patellofemoral joint overstuffing in modular, unlinked bicompartmental knees. The degree of patellofemoral (PF) overstuffing after surgery was evaluated using CT and MRI in 55 knees [47]. It was reported that modular unlinked BiKA was associated with high patient satisfaction and significant functional improvement at 5–9 years post-surgery. However, the amount of osteotomy should be carefully weighed during surgery since patient satisfaction might be affected by patellofemoral overstuffing. The ACL is the main antagonistic mechanism against the anterior drawing force caused by the contraction of the quadriceps femoris [48]. Nevertheless, if ACL was removed in TKA, symptoms might be less likely to develop because overstuffing occurs in the patellofemoral joint [49]. However, in BiKA, the intact knee ligaments render it challenging to decompress the patellofemoral joint through the anterior tibial movement. Higher patellofemoral pressure may affect postoperative symptoms in BiKA more than in TKA. Yamawaki et al. [50] demonstrated that overstuffing of the anterior femoral wall increased the contact force of the patellofemoral joint in the PFA model of dynamic computer simulations. Ogura et al. [22] found that the 2- and 5-year survival rates were 98% and 92%, respectively, which were significant improvements compared to preoperative rates. Patients were satisfied during short- and mid-term follow-up. Young and physically active patients with bicompartmental arthritis are more suitable candidates for modular unlinked BiKA. Akkawi et al. [51] systematically reviewed the results of simultaneous Bi-UKA and found no significant differences in clinical scores between Bi-UKA and UKA or between medial UKA plus patellofemoral prosthesis and TKA. The Bi-UKA group had comparable or higher scores as compared to the TKA group, and the length of hospital stay was significantly shorter in the Bi-UKA group than in the TKA group.

**Table 1:** Studies which evaluated the role of modular BCA

Author	Year	Type	Group 1, (*n*)	Group 2, (*n*)	Follow-up	Outcomes	Results
Kamath AF [15]	2014	Retrospective single cohort	BiKA, (29)	/	Minimum two-year	Functional, radiological assessment and implant survivorship	Improvement across all functional scores, mean range of motion
Uluyardimci E [17]	2019	Retrospective cohort	Inlay PFA/UKA, (49)	TKA, (49)	48–60 months	Functional assessment, complication rates, and length of hospital stay	Shorter length of hospital stay, lower complication rates, similar clinical and functional result
Benazzo F [52]	2014	Retrospective cohort	Isolated PFA, (25)	UKA + PFA, (30)	5 years	Functional assessment	Promising results at mid-term follow-up
Schrednitzki D [53]	2020	RCT	BCA, (40)	TKA, (40)	5 years	Functional assessment	No significant differences in clinical scores; significantly greater range of motion in BCA
Romagnoli S [18]	2018	Retrospective cohort	PFA, (64)	UKA + PFA, (41)	5.5 ± 1.6 years	Functional and radiological assessment	Excellent clinical and radiographic outcomes
Rossi SMP [54]	2021	Retrospective single cohort	PFA + mUKA, (45) PFA + lUK, (12)	/	nine years (6 to 13)	Functional and radiological assessment survival rate	good survival rate
Goh JKM [55]	2020	RCT	Unlinked, modular BCA, (26)	TKA, (22)	10	Functional and radiological assessment	No significant differences
Heyse TJ [37]	2010	Retrospective single cohort	UKA + PFR, (9)	/	11.8 ± 5.4 years	Functional, radiological, and satisfaction assessment	A successful approach to prevent or postpone TKA
Baba R [47]	2023	Retrospective single cohort	Modular unlinked BiKA, (55)	/	5 to 9 years	clinical assessment	High satisfaction and functional improvement were observed, but the patellar overstuffing was a problem

*BiKA* Biunicompartmental Knee Arthroplasty, *PFA* Patellofemoral Arthroplasty, *UKA* Unicompartmental Knee Arthroplasty, *TKA* Total Knee Arthroplasty, *BCA* Bicompartmental Arthroplasty, *RCT* Randomized Controlled Trial, *mUKA* Medial Unicompartmental Knee Arthroplasty, *lUKA* Lateral Unicompartmental Knee Arthroplasty, *PFR* Patellofemoral Replacement

**Table 2:** Studies which evaluated the role of monolithic BCA

Author	Year	Type	Group 1, (*n*)	Group 2, (*n*)	Follow-up	Outcomes	Results
Palumbo BT [56]	2011	Retrospective single cohort	Journey-Deuce bicompartmental knee prosthesis, (36)	/	21 months	Functional, radiological assessment, satisfaction, survival rate	Inconsistent pain relief, unacceptable functional results, and low short-term survival
Engh GA [45]	2014	RCT	Journey-Deuce bicompartmental knee prosthesis, (25)	TKA, (25)	2 years	Functional and radiological assessment	Equivalent results in clinical scores and functional testing
Aujla RS [19]	2022	Retrospective single cohort	Journey-Deuce bicompartmental knee prosthesis, (37)		11.4 years	Functional assessment	Significantly improved clinical outcomes, Comparable to other surgical arthroplasty options
Beckmann J [20]	2020	Prospective single cohort	Customized bi-compartmental knee arthroplasty, (79)	/	2 years	Functional assessment, survivorship rate	Encouraging early results
Shamdasani S [21]	2020	Retrospective single cohort	CIM BKA, (26)	/	3.2 years	Functional and radiological assessment, patellar tracking, survival rate, satisfaction, overall improvement	Clinical and patient-reported outcomes improved significantly
Ogura T [22]	2019	Retrospective single cohort	CIM BKA, (59)	/	3.8-year	Functional, radiological assessment and satisfaction survey	Significant improvement with a high level of satisfaction over short- to mid-term follow-up
Parratte S [46]	2010	Retrospective cohort	BiKA, (100)	mUKA/PFA, (77) knee	Mini 5 years	Functional assessment and prosthesis survivorship, revision rate	Relatively high failure rate

*TKA* Total Knee Arthroplasty, *BKA* Bicompartmental Knee Arthroplasty, *CIM* Customized Individually Made, *BiKA* Biunicompartmental Knee Arthroplasty, *mUKA* Medial Unicompartmental Knee Arthroplasty, *PFA* Patellofemoral Arthroplasty, *RCT* Randomized Controlled Trial

#### Which is better for primary arthroplasty, BKA or TKA?

The mid- and long-term results showed similar postoperative ROM and functional scores in the modular BCA and TKA groups (Table 3). However, a short-term follow-up displayed that BCA-M resulted in more near-natural gait and improved patient-reported outcomes (PRO + +) compared to CR-TKA after matching for age, sex, and body mass index [57]. BCA-M registered a 24% faster top walking speed, 15% longer steps, and 14% longer strides (*P* < 0.001), demonstrating greater native maximum weight acceptance (*P* < 0.001) and mid-stance forces (*P* = 0.03). Additionally, BCA-M yielded better scores in/out of a car or using public transport, rising from a chair, kneeling and getting up, sudden “give way”, episodes, and stair climbing. Two studies on monolithic prostheses exhibited that the Journey-Deuce prosthesis had a functional advantage over TKA only in the first 3 months [45, 58]. The operation time of BKA lasted longer than that of TKA [53, 59]. The calculated blood loss and length of stay were found to be significantly less in patients with BCA [17, 53]. Rossi et al. [54]compared 57 subjects receiving procedures, of which 45 patients underwent PFA with medial unicompartmental replacement and 12 received lateral unicompartmental replacement. The results showed that the 10-year survival rate of the prosthesis was 91.5%, and the outcome measures at the last follow-up were significantly improved compared with those before the operation. Sebastien et al. [4] studied the long-term functional status of PCA and retrospectively analyzed clinical and radiographic results in 84 patients (100 knees) with bi-compartmental UKA and 71 patients (77 knees) with medial UKA/PFA. A maximum follow-up of 23 years revealed that the 17-year prosthesis survival rate was 78% in the bi-compartment UKA group and 54% in the medial UKA/PFA group. In both groups, survival at 17 years was lower with revision than with conventional TKA or UKA. Jeremy et al. [55] conducted an RCT involving 48 patients with medial and patellofemoral compartment knee osteoarthritis. The subjects were divided into two groups, with 26 in PFA/UKA group and 22 in TKA. Compared with preoperative findings, the overall improvement of the two groups was not significant 10 years after surgery. Deng et al. [59] compared BKA and TKA and found that Modular BKA resulted in better joint perception, better functional recovery, and higher return-to-sport rates than TKA.

**Table 3:** Studies that evaluated BCA vs. TKA

Author	Year	Type	Group 1, (*n*)	Group 2, (*n*)	Follow-up	Outcomes	Results
Garner AJ [57]	2022	Retrospective cohort	Modular, single-stage, medial bicompartmental arthroplasty subjects, (16)	TKA, (20)	Mean months post-surgery (SD) 21 ± 18, 40.6 ± 43 Median months post-surgery (range) 12.5 (6–65), 19.5 (6–147)	Functional assessment	More near-natural gait and improved patient-reported outcomes
Uluyardimci E [17]	2019	Retrospective cohort	Inlay PFA/UKA, (49)	TKA, (49)	54 ± 4 and 54.4 ± 3.9 months	Functional and radiological assessment, complication rates, and length of hospital stay	Lower complication rates, shorter length of hospital stay, similar clinical and functional results
Schrednitzki D [53]	2020	RCT	Fixed-bearing unicompartmental knee arthroplasty, Inlay design PFJ, (40)	TKA, (40)	5 year	Functional assessment	Greater range of motion, no significant differences in clinical scores
oh JKM [55]	2020	RCT	Unlinked, modular BCA, (26)	TKA, (22)	10 years	Functional and radiological assessment	Comparable to TKA
Engh GA [45]	2014	RCT	BKA, (25)	TKA,(25)	2 years	Functional and radiological assessment	Equivalent results in clinical scores and functional testing
Morrison TA [58]	2011	Prospective cohort	BKA, (21)	TKA, (33)	2 years	Functional assessment, complication rate	Higher BKA complication rate, TKA recommended
Deng W [59]	2023	Retrospective cohort	BKA, (25)	TKA, (50)	minimum 2-year follow-up	Functional assessment	Better functional recovery, better joint perception, and higher RTS rate

*TKA* Total Knee Arthroplasty, *PFA* Patellofemoral Arthroplasty, *UKA* Unicompartmental Knee Arthroplasty, *BCA* Bicompartmental Arthroplasty, *PFJ* Patellofemoral Joint, *BKA* Bicompartmental Knee Arthroplasty, *RCT* Randomized Controlled Trial, *RTS* Return to Sport; SD: Standard Deviation

Enes et al. [17] retrospectively studied 49 inlay PFA/UKA and 49 TKA. The inlay PFA/UKA group had fewer total complications and shorter hospital stays than the TKA group. None of the patients in the inlay PFA/UKA group had radiographic evidence of progression of lateral compartment osteoarthritis according to Kellgren-Lawrence criteria. Daniel et al. [53] conducted an RCT study comparing PFA/UKA and TKA and found no significant differences in clinical scores, only the range of motion was significantly greater in PFA/UKA.

#### Which is better for revision of PKA, sBCA or TKA?

sBCA resulted in more near-natural gait and significantly higher PRO compared to revision TKA at the short-, mid-, and long-term follow-ups (Table 4). sBCA registered 16% faster top walking speed (*P* = 0.003), 13% longer steps (*P* < 0.05), and 8% longer strides (*P* < 0.05), resulting in nearer-natural weight-acceptance rate (*P* < 0.001), maximum weight-acceptance force (*P* < 0.006), mid-stance force (*P* < 0.03), contact time (*P* < 0.02), double support time (*P* < 0.009), step length (*P* = 0.003) and stride length (*P* = 0.051). sBCA yielded a better score for getting in/out of a car or using public transport, kneeling down and getting up, and preventing sudden “give up” [60]. A study by Pritchett et al. showed that results were excellent or good in 94% of sBCA patients, fair in 3%, and poor in 3%. The corresponding proportions for patients who had revision to TKA were 79%, 12%, and 9%. For patients with unilateral sBCA and contralateral revision, 81% preferred sBCA, and 19% preferred TKA. All patients reported similar or easier recovery after revision UKA-BCA than after the primary UKA [61]. Additionally, for progressive OA following PKA, sBCA showed shorter operative times and LOS and was more cost-effective [61, 62].

**Table 4:** Studies that evaluated sBCA vs TKA

Author	Year	Type	Group 1, (*n*)	Group 2, (*n*)	Follow-up	Outcome	Results
Garner AJ [60]	2023	Retrospective cohort	partial to combined partial knee arthroplasty, (23)	rTKA,(23)	17 months	Functional assessment	Nearer- natural gait and improved satisfaction
Haffar A [62]	2022	Retrospective cohort	sBiKA, (27)	rTKA, (30)	7.4 years for sBiKA; 9.7 years for rTKA	Functional assessment, operative times, length of stay, complication rates, and the need for reoperations	Equivalent survivorship, greater improvement in function, and cost-effective
Pritchett JW [61]	2022	Retrospective cohort	UKA-BCA, (73)	rTKA, (75)	15 years for UKA-BCA; 13 years for UKA-TKA	Functional assessment, and patient satisfaction	Successful treatment for disease progression following UKA

*rTKA* Revision Total Knee Arthroplasty, *sBiKA* Staged Biunicompartmental Knee Arthroplasty, *UKA* Unicompartmental Knee Arthroplasty, *BCA* Bicompartmental Arthroplasty, *UKA-BCA* Unicompartmental Knee Arthroplasty to Bicompartmental Arthroplasty, *UKA-TKA* Unicompartmental Knee Arthroplasty to Total Knee Arthroplasty

#### Complication and survivorship

DVT (1.6–3.4%), infection (1.6–3.4%), patellar subluxation (3.7–4.5%), and stiffness (4–4.7%) were the common complications of BCAs [15, 17, 53, 54, 62, 63]. Survivorship of BCAs was 95.1–96% at 5 years [23, 64]. 91.5% at 10 years [54] and 54–58% at 17 years [23, 46]. Compared with TKAs, BCAs had a higher revision rate within 1 year (OR, 1.761; 95% CI, 1.150–2.568; *P* = 0.006) and 2 years (OR, 2.097; 95% CI, 1.547–2.775; *P* < 0.001). However, BCA was associated with a lower 90-day complication rate (OR: 0.483; 95% CI: 0.397–0.584; *P* < 0.001), including major complications [OR: 0.515; 95% CI: 0.303–0.812; *P* = 0.008], such as renal failure (OR: 0.224; 95% CI: 0.055–0.588; *P* = 0.010), anemia (OR:0.267; 95% CI: 0.183–0.374; *P* = 0.001), blood transfusion (OR: 0.473; 95% CI: 0.259–0.787; *P* = 0.008), pneumonia (OR: 0.122; 95% CI: 0.007–0.539; *P* = 0.034), heart failure (OR: 0.292; 95% CI: 0.102–0.653; *P* = 0.008), and urinary tract infection (OR: 0.652; 95% CI: 0.421–0.960; *P* = 0.041) [63]. Revisions of the PKA and sBCA demonstrated significant functional improvement, with comparable long-term survival rates [61, 62]. Given these outcomes, sBiKA is a safe, effective, and economical alternative to rTKA for treating progressive OA after PKA. Nevertheless, further follow-up is required to determine whether sBiKA is a durable treatment option.

### Robotic approach

In robotic arm-assisted (RA) arthroplasty, the surgeon can modify the implant position and component size to fit the patient’s knee before osteotomy. The ACL and bone spur were preserved, allowing potential benefits while minimizing early failure from alignment issues. A few studies focused on the accuracy of RA BCA, with favorable functional outcomes, high survival rates, and excellent satisfaction at mid-term follow-up. Burger et al. [65] suggested that in the hands of experienced surgeons, RA surgery can achieve results comparable to the traditional UKA technique. Robotic surgery results in more reliable lower limb alignment, improved prosthesis positioning, and accurate restoration of ligament balance over the entire range of motion.

Gaudiani et al. [64] reviewed a single-center prospective cohort of 50 patients (53 knees) who underwent BiKA (postoperative patellofemoral and medial compartment follow-ups at 5 and 7 years). At 7 years of follow-up, 76% were satisfied, 13% were neutral, and 11% were dissatisfied. The mean KSS-FS was 80.5 ± 15.8, with 82% reporting walking more than 10 blocks, 89% walking without support, 100% being able to walk up and down stairs, and 61% requiring a handrail. The study reported good survival and functional outcomes with robotic arm-assisted BiKA and high satisfaction rates.

Watanabe et al. [31] summarized 13 patients who underwent 15 multi-compartmental knee arthroplasties using haptic robotic-assisted bone preparations. To restore or maintain near-normal knee kinematics, it is necessary to preserve intact structures and compartments as much as possible. At maximum flexion kneeling, the knee kinematics showed femoral external rotation and posterolateral condylar translation. All knees exhibited femoral external rotation and posterior condylar flexion translation during step movement. The medial UKA and PFA had the highest degrees of femoral external rotation and posterior translation, while the Bi-UKA had the least. Future registry-based randomized studies are warranted to compare the outcomes of RA treated using conventional Bi-UKA techniques.

## Conclusions

BCA is beneficial for patients with bicompartmental or tricompartmental arthritis or those with progression to other compartments after UKA. Compared to TKA, it can reduce trauma, accelerate recovery, and achieve better sports ability. Studies differed on complications and revision rates for BCA. The revision rate of the modular prosthesis was similar to that of TKA. However, the failure rate of repair with SN Deuce was significantly higher than that with TKA. Both short- and long-term follow-up data showed significant functional improvement after BCA with both modular and monolithic prostheses. Combined with the revision rate, the long-term data favor modular prosthesis. BCA is still under development, and future research can go along a great many exciting directions. However, more clinical studies are needed to explore and validate its benefits. The ultimate goal is to optimize function and improve patient satisfaction.

## Data Availability

The datasets generated and analyzed in this study are available from the corresponding author upon reasonable request.

## Abbreviations

BCA: Bicompartmental arthroplasty
TKA: Total knee arthroplasty
OA: Osteoarthritis
UKA: Unicompartmental knee arthroplasty
PKA: Partial knee arthroplasty
CPKA: Combined partial knee arthroplasty
BCA-M: Medial bicompartmental arthroplasty
BCA-L: Lateral bicompartmental arthroplasty
Bi-UKA: Biunicondylar arthroplasty
PFA: Patellofemoral arthroplasty
CIM: Customized individually made
OTS: Off-the-shelf
CR-TKA: Cruciate-Retaining total knee arthroplasty
ACL: Anterior cruciate ligament
A-P: Anterior-posterior
MCL: Medial collateral ligament
sBCA: Staged bicompartmental knee arthroplasty
PRO: Patient-reported outcomes
ROM: Range of motion
KOOS: Knee injury and osteoarthritis outcome score
KSS: Knee society score
LOS: Length of stay
PF: Patellofemoral
TF: Tibiofemoral
RA: Robotic arm-assisted
BiKA: Biunicompartmental knee arthroplasty
KSS-FS: Knee society score-function score
